# Neuroprotective Effect of *Protaetia brevitarsis seulensis*’ Water Extract on Trimethyltin-Induced Seizures and Hippocampal Neurodegeneration

**DOI:** 10.3390/ijms22020679

**Published:** 2021-01-12

**Authors:** Sueun Lee, Young Hye Seo, Jun Ho Song, Wook Jin Kim, Ji Hye Lee, Byeong Cheol Moon, Mary Jasmin Ang, Sung Ho Kim, Changjong Moon, Jun Lee, Joong Sun Kim

**Affiliations:** 1 Herbal Medicine Resources Research Center, Korea Institute of Oriental Medicine, Naju 58245, Korea; leese@kiom.re.kr (S.L.); wnsl1118@kiom.re.kr (Y.H.S.); songjh@kiom.re.kr (J.H.S.); ukgene@kiom.re.kr (W.J.K.); 2jh@kiom.re.kr (J.H.L.); bcmoon@kiom.re.kr (B.C.M.); 2 College of Veterinary Medicine and BK21 Plus Project Team, Chonnam National University, Gwangju 61186, Korea; 166371@jnu.ac.kr (M.J.A.); shokim@chonnam.ac.kr (S.H.K.); moonc@chonnam.ac.kr (C.M.)

**Keywords:** *Protaetia brevitarsis seulensis* larva, trimethyltin, seizure, hippocampal neurodegeneration, antioxidant, chemical profiling

## Abstract

This study aimed to investigate whether the *Protaetia brevitarsis seulensis* (PB)’ water extract (PBWE) ameliorates trimethyltin (TMT)-induced seizures and hippocampal neurodegeneration. To investigate the potential neuroprotective effect of the PBWE in vitro, a lactate dehydrogenase (LDH) assay was conducted in TMT-treated primary cultures of mouse hippocampal neurons. In TMT-treated adult C57BL/6 mice, behavioral and histopathological changes were evaluated by seizure scoring and Fluoro-Jade C staining, respectively. In our in vitro assay, we observed that pretreating mice hippocampal neuron cultures with the PBWE reduced TMT-induced cytotoxicity, as indicated by the decreased LDH release. Furthermore, pretreatment with the PBWE alleviated seizures and hippocampal neurodegeneration in TMT-treated mice. The antioxidant activity of the PBWE increased in a dose-dependent manner; moreover, pretreatment with the PBWE mitigated the TMT-induced Nrf2 stimulation. In addition, six major compounds, including adenine, hypoxanthine, uridine, adenosine, inosine, and benzoic acid, were isolated from the PBWE, and among them, inosine and benzoic acid have been confirmed to have an essential antioxidative activity. In conclusion, the PBWE ameliorated TMT-induced toxicity in hippocampal neurons in both in vitro and in vivo assays, through a potential antioxidative effect. Our findings suggest that the PBWE may have pharmacotherapeutic potential in neurodegenerative diseases such as seizures or epilepsy.

## 1. Introduction

Trimethyltin (TMT), a neurotoxic organotin, induces severe neurodegeneration and neuronal death in the central nervous system, particularly in the hippocampus [1]. Clinical features of TMT intoxication include headache, cognitive impairment, insomnia, and tonic-clonic seizures [2,3]. These neurological and behavioral signs and symptoms are clinically similar to those of temporal lobe epilepsy, which is common in adults [4,5]. Thus, TMT-intoxicated animal models may be successfully used as a model for limbic system neurodegeneration or temporal lobe epilepsy [6]. Various mechanisms have been investigated to understand the etiopathology of TMT-induced neurodegeneration; the accumulation of excessive reactive oxygen species (ROS) has been proposed as one of the main underlying mechanisms [7,8].

Traditionally, insect bodies, larvae, eggs, eggshells, and their secretions have been used as sources of food or medicine. The larvae of the *Protaetia* Burmeister, 1842, called “Gum-Beng-I” in Korea, are known oriental medicinal products [9]. In particular, among the six species found in Korea [10], the larvae of *P. brevitarsis seulensis* (Kolbe), 1886 (PB), a white-spotted flower chafer, have been widely used in traditional medicine to treat various diseases, including hepatitis, liver cirrhosis, breast cancer, tetanus, thrush, and toxic epilepsy [11,12]. According to the Dong Ui Bo Gam, an ancient classical text of Korean medicine, the PB larvae were especially useful in the treatment of seizures [13]. However, as of today, no study has investigated the potential protective effect of PB larvae on animal models of seizures or epilepsy.

Reports on the proximate composition of PB larvae have stated that it contains high contents of proteins and fats, especially a high proportion of unsaturated lipids, such as oleic and palmitoleic acids [11]. Furthermore, some studies have revealed the presence of secondary metabolites in the ethanol extract of PB larvae, including a quinoxaline, indole alkaloids, indoles, dopamines, and diketopiperazine derivatives [12,14], as well as some volatile compounds [11]. However, there is no information on the composition of the PB larvae water extract (PBWE), even though Asian medical products have traditionally been extracted using boiling water [15].

The aim of the present study was to determine whether the PBWE was useful in ameliorating TMT-induced seizures and whether it had any potential neuroprotective effect on hippocampal neurodegeneration. We also aimed to isolate the major components of the PBWE, and confirm which component could exhibit a possible involvement in the neuroprotective effect. 

## 2. Results

### 2.1. Morphological Identification and Genetic Analysis

The morphological characteristics of the dried PB larvae are observed. PB larvae are ellipsoid in shape, brownish in color, and glossy. The size of the head capsule ranged from 1.0–1.3 mm in length and 4.2–4.6 mm in width.

**Figure 1:**
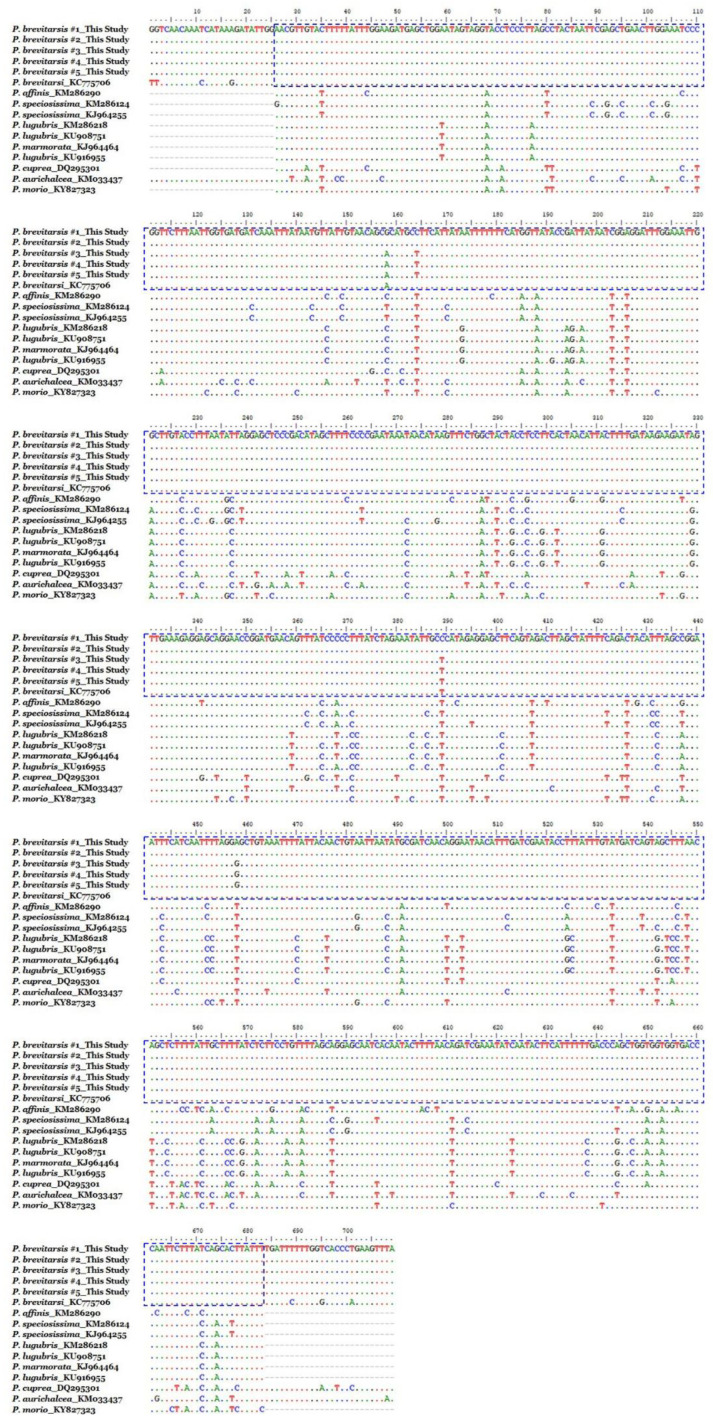
Comparative analysis of the cytochrome oxidase subunit 1 DNA barcode sequences between *Protaetia brevitarsis* (PB) samples and *Protaetia* species registered in GenBank. Dots (.) and dashes (-) indicate identical nucleotides and gaps with “*P. brevitarsis* #1_This study” respectively, when compared with corresponding cytochrome oxidase subunit 1 sequence of PB larvae samples used in this study, to maximize sequence alignment presentation. The boxes indicate sequences identical with that of the *Protaetia brevitarsis* (accession no. KC775706). Each nucleotides are marked in different colors: green, A; blue, C; red, T; black, G.

The five randomly selected PB larvae were assessed for genetic species identification. A total of 16 cytochrome oxidase subunit 1 (COI) sequences including those from PB and its closely related species were used for comparative analysis based on multiple sequence alignment (Figure 1). The five samples used in this study were confirmed as authentic *P. brevitarsis*, with different COI sequences when compared to the closely related species. Intraspecies sequence variation in five *P. brevitarsis* samples was shown at four nucleotide positions, which were 158 (G/A), 164 (C/T), 389 (C/T), and 458 (A/G). Thus, intraspecies sequence variability was about 0.34% in *P. brevitarsis*. 

### 2.2. The PBWE Ameliorated TMT-Induced Neurotoxicity in Hippocampal Cultured Neurons

**Figure 2:**
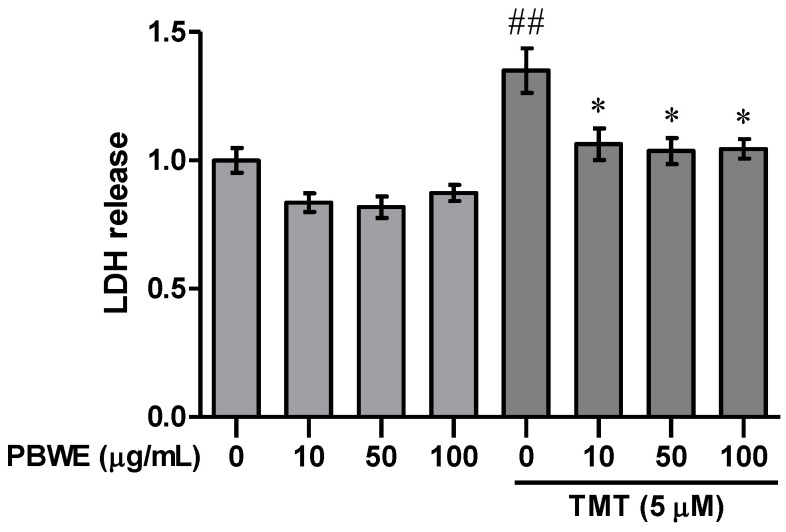
Protective effect of the *Protaetia brevitarsis seulensis’* water extract (PBWE) on trimethyltin (TMT)-induced cytotoxicity. The PBWE treatment (10–100 μg/mL) was confirmed to reduce the cytotoxic activity of 5 μM TMT on hippocampal neurons in the lactate dehydrogenase (LDH) assay. Values are reported as mean ± SE. ## *p* < 0.01 vs. saline-treated group, * *p* < 0.05 vs. TMT-alone treated group.

We performed the lactate dehydrogenase (LDH) release assay to confirm whether the PBWE treatment alleviates TMT-induced injury in hippocampal cells at days in vitro (DIV) 7. We found that treatment with 5 μM TMT alone increased LDH release from cultured hippocampal neurons compared to that in vehicle controls at 24 h after treatment (*n* = 3 cultures per condition, *p* < 0.01; Figure 2); moreover, pretreatment with the PBWE (10–100 μg/mL) significantly inhibited TMT-induced cytotoxicity (*n* = 3 cultures per condition, *p* < 0.05; Figure 2).

### 2.3. The PBWE Alleviated TMT-Induced Seizures in the In Vivo Model

**Figure 3:**
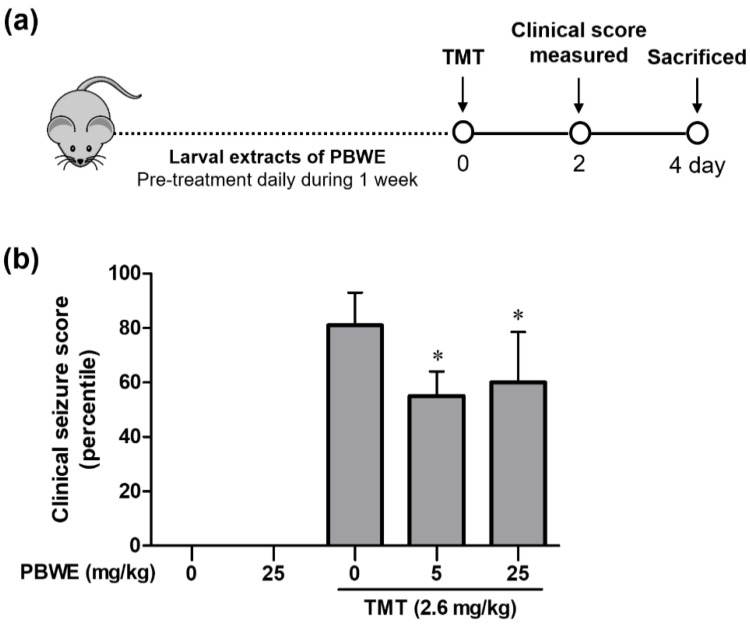
Protective effect of the *Protaetia brevitarsis seulensis*’ water extract (PBWE) on seizure symptoms in trimethyltin (TMT)-treated mice. (**a**) Schematic diagram showing the timeline of drug administration, behavioral tests, and sacrifice. (**b**) PBWE pretreatment ameliorated TMT-induced seizure behaviors on day 2 (*n* = 10 mice per group). Values are reported as median of percentile + interquartile. * *p* < 0.05 vs. TMT-alone treated group.

According to previous studies, TMT-induced seizures are most significant on day 2 post-treatment, decreasing afterwards [16,17,18]. Therefore, we compared TMT-induced seizures between groups on day 2 after TMT treatment (Figure 3a). TMT-injected mice presented systemic tremor and spasmodic gait (Figure 3b). However, PBWE + TMT-treated mice showed a significant decrease in seizure scores, mainly due to weak and systemic tremors, compared to the TMT-alone treated mice (*n* = 10 mice per group, * *p* < 0.05; Figure 3b). Furthermore, while two mice died in the latter group, only one mouse died in the 5 mg/kg and none died in the 25 mg/kg PBWE + TMT groups (data not shown).

### 2.4. Pretreatment with the PBWE Attenuated Hippocampal Neurodegeneration in TMT-Treated Mice

**Figure 4:**
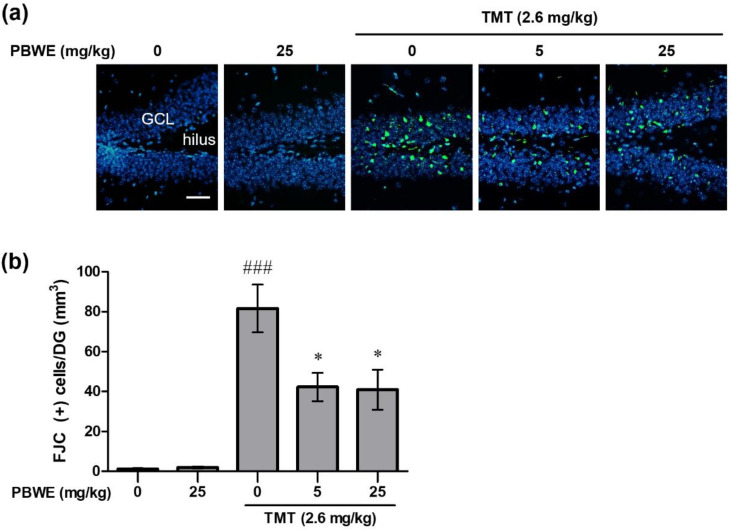
Protective effect of the *Protaetia brevitarsis seulensis’* water extract (PBWE) on the number of Fluoro-Jade C (FJC)-positive degenerating neurons in the dentate gyrus (DG) of hippocampus on day 4 post-trimethyltin (TMT)-treatment. (**a**) Representative images (200×) show degenerating FJC-stained cells (green color) in the saline-treated control, TMT-treated control, and PBWE-pretreated TMT groups. (**b**) Graph depicts the number of FJC-positive cells per DG, in the hippocampus sections (*n* = 4 in each group). Values are reported as mean ± SE. ### *p* < 0.001 vs. saline-treated group, * *p* < 0.05 vs. TMT alone-treated group. Scale bar represents 40 μm. GCL, granular cell layer.

Figure 4a shows the representative photos of Fluoro-Jade C (FJC)-stained degenerating neurons in the hippocampal dentate gyrus (DG) of each group, especially the granular cell layer and subgranular zone. We found that the number of FJC-positive cells in the TMT-treated mice markedly increased in comparison to that in the vehicle control mice (### *p* < 0.001 vs. saline-treated group; Figure 4b). Furthermore, the number of degenerating neurons was significantly diminished in the PBWE + TMT-treated mice compared to that in TMT-alone treated mice (* *p* < 0.05 vs. TMT alone-treated group; Figure 4b).

### 2.5. The PBWE Scavenged Free Radicals in the 2,2-Diphenyl-1-Picryl-Hydrazyl-Hydrate (DPPH) Assay, and Pretreatment with the PBWE Reduced the Protein Expression of Nuclear Factor Erythroid 2-Related Factor 2(Nrf2) in the In Vivo Model

**Figure 5:**
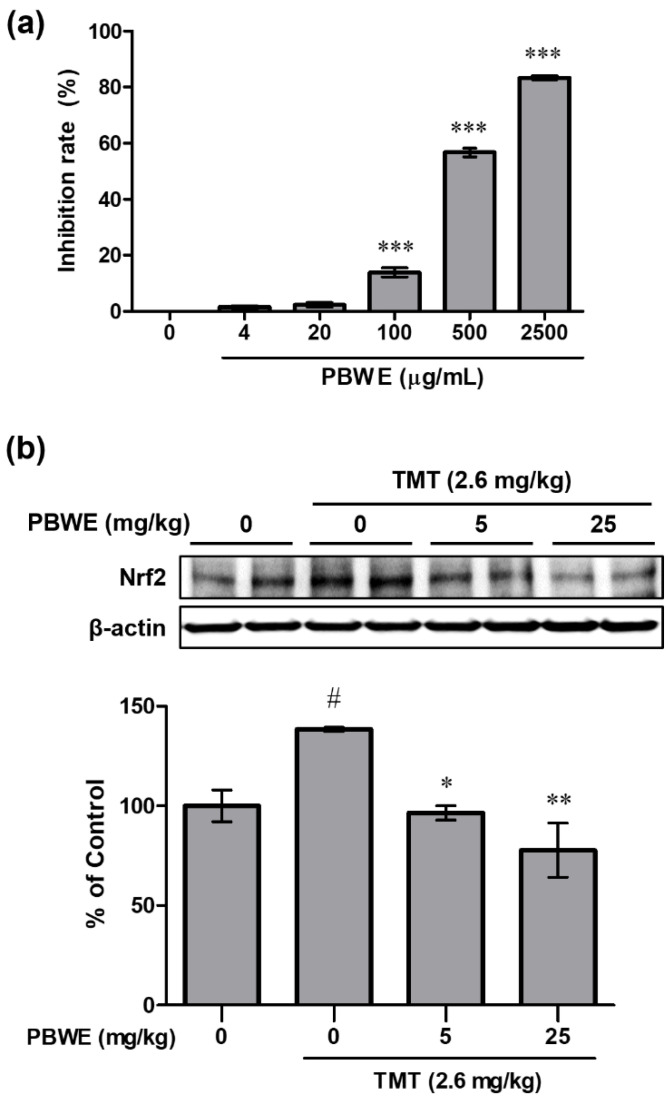
Antioxidative potential of the *Protaetia brevitarsis seulensis’* water extract (PBWE) and protein expression of nuclear factor erythroid 2-related factor 2 (Nrf2) in the PBWE and trimethyltin (TMT)-treated mouse hippocampus. (**a**) In the 2,2-diphenyl-1-picryl-hydrazyl-hydrate assay, the PBWE markedly scavenged the free radicals in a dose-dependent manner. *** *p* < 0.001 vs. 0 μg/mL of PBWE-treated group. (**b**) PBWE pretreatment abrogated the increased protein expression of Nrf2 on day 4 post-TMT treatment. Values are reported as mean ± SE. # *p* < 0.05 vs. saline-treated group, * *p* < 0.05 and ** *p* < 0.01 vs. TMT-alone treated group.

To evaluate the antioxidative potential of the PBWE, we measured its inhibition rate (%) against DPPH radicals. The inhibition rate significantly increased, compared to that in the blank control group, in a dose-dependent manner above the dose of 100 μg/mL of the PBWE (*n* = 4 per group, *** *p* < 0.001; Figure 5a). The inhibition rate in the highest dose (2500 μg/mL) of the PBWE was 83.35%, and the half inhibitory concentration (IC_50_) of the PBWE was 403.3 μg/mL as estimated using by the DPPH assay.

In addition, we examined the protein expression of Nrf2 in the mouse hippocampus on day 4 post-TMT treatment. The level in TMT alone-treated group significantly increased compared to that in the saline-treated group (### *p* < 0.001), but the expression level was evidently reduced in the PBWE + TMT-treated groups (*n* = 3 mice per group, * *p* < 0.05 and ** *p* < 0.01; Figure 5b) compared to that in the TMT alone-treated mice.

### 2.6. Ultra-Performance Liquid Chromatography (UPLC) Chemical Profile of the PBWE and Antioxidative Activity of Isolated Compounds

**Figure 6:**
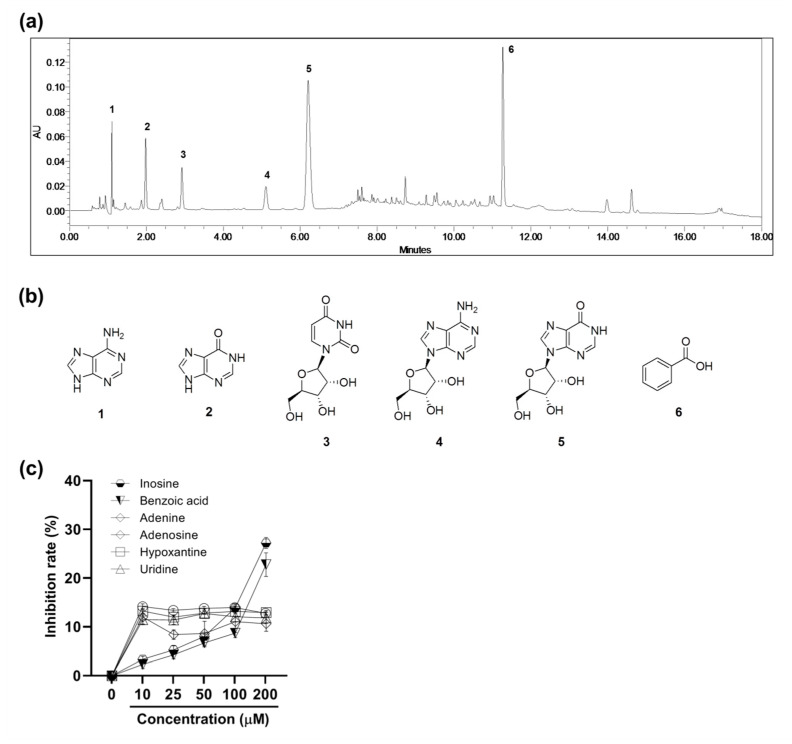
The ultra-performance liquid chromatography (UPLC) chemical profile of the *Protaetia brevitarsis seulensis’* water extract and antioxidative activity of isolated compounds. (**a**) UPLC chromatogram of the extract with detection at ultra-violet 254 nm. (**b**) Chemical structures of compounds including adenine (**1**), hypoxanthine (**2**), uridine (**3**), adenosine (**4**), inosine (**5**), and benzoic acid (**6**). (**c**) The free radical scavenging activity of the isolated six compounds in the 2,2-diphenyl-1-picryl-hydrazyl-hydrate assay. Values are reported as mean ± SE.

A chemical investigation of the PBWE led to the isolation of six major compounds by chromatographic separation methods. The structures of the isolated compounds were identified through interpretation of their spectral data including one and two-dimensional nuclear magnetic resonance (NMR) and comparison with reported values. The isolated compounds were identified as adenine (**1**) [19], hypoxanthine (**2**) [19], uridine (**3**) [20], adenosine (**4**) [21], inosine (**5**) [21], and benzoic acid (**6**) [22]. All compounds except benzoic acid (**6**) were purine or pyrimidine derivatives. The UPLC chemical profile of the PBWE and chemical structures of each peak are shown in Figure 6. Compounds **1**−**6** were detected at 1.099, 1.980, 2.923, 5.106, 6.205, and 11.269 min, respectively. 

To examine the antioxidative activity of the isolated compounds, which could affect that of PBWE, we identified the free radical scavenging rate (%) in the DPPH assay (*n* = 5–6 per group; Figure 6c). The inhibition rates of inosine and benzoic acid significantly increased in a dose-dependent manner above the dose of 10 μM and 50 μM, respectively, compared to that in the blank control. IC_50_ of inosine and benzoic acid were 338.3 μM and 472.0 μM, respectively. Meanwhile, in case of the other compounds, the inhibition rates did not follow a dose-dependent manner, although all values above 10 μM were significantly higher than that of the blank control. 

## 3. Discussion

Insects have been widely and traditionally used as medication due to their pharmacological effects and easy accessibility; however, misuse of the said medicinal products because of adulterants or counterfeits may result in severe problems for patients. Thus, accurate identification of medicinal products is essential for research and clinical application. To this end, various methods have been recently used [23]; in the present study, we used morphological and DNA analysis methods and confirmed the samples as authentic PB, the analytical data of which are consistent with those in previous reports [10,24]. Although there are some variations between the COI sequence of the five PB samples, the low intraspecies variation rate of the COI region located in the mitochondrial genome inherited as a haplotype was considered as an individual variation [25].

Although PB larvae have been broadly used in oriental medicine to treat several diseases, few studies have been conducted on its protective effect in neurodegenerative diseases. Thus, we selected the TMT-intoxicated models for investigating the neuroprotective effects of PB larvae. TMT induces clinical symptoms of central nervous system, such as learning impairment and tonic-clonic seizure [26]. The toxin also leads to neurodegeneration in the limbic system especially the hippocampus, via various events, including oxidative stress, glutamate excitotoxicity, neuroinflammation, intracellular calcium overload, impaired neurotransmission, and mitochondrial dysfunction [2,6,27]. Mice intoxicated by TMT exhibit seizure behavior and histopathological damage maximally on day 2, which persist until about 8 days after TMT treatment [28], but the clinical symptoms and the accompanied hippocampal lesions are recovered over time through spontaneous neuroregenerative processes [16,29]. In this study, we performed an in vitro experiment that showed that pretreatment with the PBWE reduced TMT-induced neuronal death in primary cultured hippocampal neurons. Furthermore, in our in vivo model, oral pretreatment with the PBWE decreased death rate and significantly reduced the seizure severity on day 2 post-TMT treatment. Additionally, pretreatment with the PBWE substantially decreased the number of FJC-positive degenerating neurons in TMT-treated hippocampal DG, consistent with the improvement in clinical symptoms. Thus, we may conclude that pretreatment with the PBWE exhibited neuroprotective effects, which resulted in reducing seizure severity and neuronal cell death in the TMT-induced neurotoxicity model.

Uncontrolled ROS accumulation and oxidative stress in the brain may be implicated in the progression of various neurodegenerative diseases [30,31]. In TMT-induced neurotoxicity, oxidative stress is also thought to be one of the main mechanisms underlying seizures and neurodegeneration. Previous studies have reported that TMT treatment induces the production of reactive oxygen and nitrogen species, protein carbonyl (a marker of protein peroxidation), malondialdehyde, and 4-hydroxynonenal (markers of lipid peroxidation) in both in vivo and in vitro models [7,32,33]. This oxidative stress condition could affect behavioral abnormalities and homeostasis imbalance of the antioxidant system, and consequently lead to neuronal cell death in TMT-intoxicated animals [7]. Further, endogenous compensatory antioxidative responses are simultaneously activated for spontaneous recovery in various neurodegenerative states, such as TMT intoxication. Earlier studies have shown that Nrf2 signaling, a major transcriptional activator of genes coding enzymatic antioxidants such as superoxide dismutase, glutathione peroxidase, and catalase, is activated in the hippocampus of TMT-treated mice [34,35]. We also observed the increased expression of Nrf2 in response to TMT-induced damage, which is consistent with previous studies; however, pretreatment with the PBWE reduced the expression of Nrf2 in the hippocampus of TMT-treated mice. Moreover, we identified that the PBWE has free radical scavenging potential as a non-enzymatic antioxidant, similar to that of nitric oxide and ROS-removal capacities of the PB larvae that has been reported in other studies [36,37]. Previous publications have reported that the administration of non-enzymatic antioxidants, including vitamin C, lycopene, and N-acetyl cysteine (NAC) alleviate TMT neurotoxicity by decreasing levels of the biomarkers of oxidative stress and consequently neuronal death [33,38,39]. Gallorini, et al. [40] showed that Nrf2 expression was decreased in the presence of NAC in 2-hydroxyethyl methacrylate-induced oxidative stress state when compared to that in subjects who had not received NAC. The underlying hypothesis was that NAC participates in the scavenging of ROS and supports the synthesis of glutathione, one of the non-enzymatic antioxidants. Similarly, the drastic decrease of heme oxygenase-1 (HO-1; Nrf2-regulated downstream gene) expression in the presence of NAC implicated that its antioxidant activity was effective enough to modulate the cellular redox balance apart from that of the enzymatic antioxidants produced by the Nrf2/HO-1 pathway in bilirubin-induced oxidative stress state of neuroblastoma cells [40,41]. Therefore, we thought that the PBWE could also present a similar antioxidant capacity with regard to the activation of Nrf2 signaling, and the antioxidative effects of the PBWE could ameliorate TMT-induced seizures and neurodegeneration.

With the recent approval of PB larvae as a food ingredient by the Ministry of Food and Drug Safety of Korea, the commercial consumption of the PB larvae has increased [12]. However, little is known about the chemical composition of PB larvae and the pharmacological effects of its components. In previous chemical studies, some compounds have been reported to be present in the ethanol extract of PB larvae, including 5-hydroxyindolin-2-one and (1*R*,3*S*)-1-methyl-1,2,3,4-tetrahydro-*β*-carboline-3-carboxylic acid, which exhibit anti-thrombotic activity [12,14,42]. As there is no research on the chemical constituents of the water extract of PB larvae, we have, to the best of our knowledge, isolated, identified, and reported for the first time six major components of the PBWE (**1**−**6**) including four purine derivatives (e.g., adenine, hypoxanthine, adenosine, and inosine), a pyrimidine derivative (uridine), and a benzoic acid. Purines and pyrimidines are heterocyclic aromatic compounds composed of carbon and nitrogen. Their derivatives play crucial roles in organisms as nucleoside/nucleotide precursors that constitute building blocks of DNA and RNA. They are known to have several biological activities such as antimicrobial, antitumor, antiviral, and antiproliferative effects [43]. Of these, uridine (**3**) and inosine (**5**), two of the major compounds in the PBWE, have been reported to exhibit anticonvulsant and antiepileptic effects by modulating the function of gamma-aminobutyric acid receptors [44,45]. Sodium benzoate, produced by the neutralization of benzoic acid (**6**), has also been shown to alleviate seizures in nonketotic hyperglycinemia [46]. In addition, uridine (**3**), inosine (**5**), and benzoic acid (**6**) have also been reported to have antioxidant activities [47,48,49]. Similarly, in the present study, we confirmed the antioxidant activity of inosine (**3**) and benzoic acid (**6**) especially among the isolated compounds. Based on the previous and present studies, we further support the idea that the components of the PBWE might present protective effects against TMT-induced seizures and neurodegeneration. However, further studies on the correlation between these components and their neuroprotective effects as well as their underlying mechanism are needed.

## 4. Materials and Methods 

### 4.1. Medicinal Materials: PB Larvae

Dried PB larvae were purchased in medicinal markets from commercial suppliers (Kwang Myung Dang Co., Ulsan, Korea). The identity of the samples (manufacturer’s No. K2281201707) was macroscopically confirmed and then deposited in the Korean Herbarium of Standard Herbal Resources (Index Herbariorum code KIOM) at KIOM, Naju, Korea (medicinal ID: 2-18-0111).

### 4.2. Morphological Identification and DNA Barcoding of PB Larvae

Since medicinal insects are often sold in a mixture of various species, it is imperative to accurately identify the species of interest. Therefore, to identify the species of PB larvae, morphological characteristics were observed using a stereomicroscope (Olympus SZX16; Olympus, Tokyo, Japan). Images were captured using a digital camera (Olympus DP21; Olympus) attached to the microscope and cellSens Standard software 2 (Olympus). Measurements and macroscopic observations about the shape, color and size of 20 randomly selected PB larvae were recorded.

Moreover, to additionally confirm that the specimens belonged to our species of interest, we amplified and sequenced the mitochondrial COI DNA barcode regions, a universal DNA barcode used for identifying animal species, from five randomly selected larvae. Then, we compared the sequence identity with other COI sequences registered in the GenBank using Basic Local Alignment Search Tool (BLAST) analysis [50]. Furthermore, to confirm the sequence identity and species of the sampled larvae, comparative sequence analysis was carried out between the COI sequences of the five samples and other closely related insect species registered in the GenBank, including *Protaetia brevitarsis* (KC775706), *P. affinis* (KM286290), *P. speciosissima* (KM286124 and KJ964255), *P. lugubris* (KM286218, KU908751, and KU916955), *P. marmorata* (KJ964464), *P. cuprea* (DQ295301), *P. aurichalcea* (KM033437), and *P. morio* (KY827323).

### 4.3. Extraction

A total of 887.4 g of dried PB larvae were ground, and the components were extracted with distilled water (15 L) under reflux at 100 ± 2 °C for 3 h. The extract was filtered, evaporated in an air vacuum, and freeze-dried to obtain the crude extract (242.8 g, 27.4%), which was then stored at −20 °C for this study.

### 4.4. Primary Hippocampal Cell Culture and Treatment with the PBWE

A primary culture of hippocampal neurons was prepared following previously described methods [16,51]. Briefly, hippocampi were dissected from C57BL/6 mice pups at 17–18 gestational days; then, neurons were isolated and subsequently cultured in vitro, following standard procedures. The cells were seeded at a density of 5 × 10^5^ cells/well in poly-D-lysine hydrobromide (150 μg/mL; Sigma-Aldrich, St. Louis, MO, USA)-coated 24-well plates with B27 (Invitrogen, Carlsbad, CA, USA)-supplemented Neurobasal A (Invitrogen) containing 100 units/mL of penicillin, 0.1 mg/mL of streptomycin, and 0.5 mM of glutamine (Invitrogen). All cultures were maintained at 37 °C and 5% CO_2_. The cultures were then treated with a 5 μM TMT solution at 7 DIV and assessed 24 h after. To evaluate the cytoprotective effect of the PBWE on the TMT-induced damage on mature hippocampal neurons, the PBWE (0–100 μg/mL) was added 1 h before the TMT treatment (*n* = 3 cultures per condition).

### 4.5. Cytotoxicity Examination

Cytotoxicity was examined using a LDH release assay (*n* = 3 per group). A LDH cytotoxicity assay kit from Biovision (Mountain View, CA, USA) was used according to the manufacturer’s recommendations. The optical density values were quantified by measuring absorbance at a wavelength of 450 nm, using a microplate reader (Emax, Molecular Devices, Sunnyvale, CA, USA) [16].

### 4.6. Animal Models, Drug Treatments, and Seizure Scoring

Specific pathogen-free male C57BL/6 mice (8 weeks, 20–22 g) were purchased from Orient Bio, Inc. (Seoul, Korea), were maintained and housed in standard cages and exposed to a 12:12 hour light/dark cycle (7 AM:7 PM) at room temperature (25 °C) with food and water ad libitum in a specific-pathogen-free facility. The animals were used after a one-week period of quarantine and acclimatization. All experimental protocols in this study were approved by the Institutional Animal Care and Use Committee of Chonnam National University (CNU IACUC-YB-2018-69, 1 August 2018), and the animals were cared for in accordance with the NIH Guide for the Care and Use of Laboratory Animals.

TMT (2.6 mg/kg; Wako, Osaka, Japan), dissolved in 0.9% saline, was intraperitoneally administered to mice. The vehicle control mice were injected with 0.9% saline. To estimate the effects of the PBWE on TMT-induced neuronal injury, the PBWE (5 or 25 mg/kg) or vehicle (0.9% saline) were perorally administered to mice daily for one week, before being administered TMT (*n* = 10 mice per group). Seizure behavior tests were performed in a bright box (40 × 40 cm, 250 lux) at about 8 AM daily for 4 days after TMT treatment. Behavioral changes were evaluated using a scoring system as follows: score 1 (aggression), score 2 (weak tremor), score 3 (systemic tremor), score 4 (tremor and spasmodic gait), and score 5 (death) [17,52]. All animals were sacrificed by decapitation on day 4 post-TMT treatment.

### 4.7. FJC Staining

To observe TMT-induced neurodegenerative changes in the hippocampus, the brain hemisphere samples were embedded in paraffin wax after fixation with a 4% paraformaldehyde solution. The hemispheres were sectioned approximately 1.44–1.56 mm from the median border. Next, 4 μm thick sagittal sections were prepared and then subjected to FJC staining according to a previously described method (*n* = 4 mice per group) [18,53]. In brief, sections were first transferred to a solution of 0.06% potassium permanganate, and then to a 0.0001% FJC staining solution (Millipore, Temecula, CA, USA). FJC is a highly anionic and acidic marker with an affinity for degenerating neurons. After FJC staining, sections were counterstained with 4′,6-diamidino-2-phenylindole·dihydrochloride (Thermo Fisher Scientific, Waltham, MA, USA) to label nuclei, before being mounted onto microscope slides. FJC-stained sections were examined using immunofluorescence microscopy on a BX-40 apparatus (Olympus) with a mounted eXcope X3 digital camera (DIXI Optics, Daejeon, Korea).

### 4.8. DPPH Radical Scavenging Assay

Free radical scavenging activity was assessed using a modified version of the method proposed by Cheng, et al. [54] (*n* = 4–6 per group). In brief, 100 μL of sample extracts (4, 20, 100, 500, 2500 μg/mL) or isolated compounds (10, 25, 50, 100, 200 μM) dissolved in 50% ethanol were added to 100 μL of 0.2 mM DPPH (Fisher Scientific, Leicestershire, UK) solution, reaching a final concentration of 0.1 mM DPPH. Each reaction mixture was shaken for five seconds and left to stand for 30 min in the dark at 25 °C. The absorbance of each mixture was measured at 515 nm against a blank (50% ethanol) using a SpectraMax i3x Microplate spectrophotometer (Molecular devices). The inhibition rate of DPPH radicals was calculated as follows:

DPPH inhibition rate (%) = (1 − [A_sample_ − A_blank_]/[A_control_ − A_blank_]) × 100

### 4.9. Western Blotting

Dissected hippocampi were immediately immersed individually in a lysis buffer and sonicated for 10 s (*n* = 3 mice per group). Sodium dodecyl sulfate (SDS) sample buffer (4×) was added to each homogenized sample, and the samples were heated at 100 °C for 10 min. Immunoblotting was performed as described previously [55]. Briefly, the resolved proteins were separated with SDS-polyacrylamide gel electrophoresis (Bio-Rad, Hercules, CA, USA) and transferred onto membrane by using Trans-Blot Turbo Transfer System (Bio-Rad). The membrane was incubated with rabbit anti-Nrf2 (1:1000; Cell Signaling, Danvers, MA, USA) overnight at 4 °C. After extensive washing and incubation with secondary antibodies for 2 h at room temperature, signals were developed using a chemiluminescence kit (SuperSignal^®^ West Pico PLUS; Thermo Fisher Scientific) and read on a ChemiDoc MP Imaging System (Bio-Rad). To quantify Nrf2 expression, the membrane was re-probed with an antibody for β-actin (1:10,000; Sigma-Aldrich). Several exposure times were used to obtain signals within the linear range, and the bands were quantified using the ImageJ software (NIH, Bethesda, MD, USA).

### 4.10. Isolation of the PBWE Components

The PBWE (50.0 g) was separated using a Diaion HP-20 gel (Supelco, Bellefonte, PA, USA) in two 340 g scale Biotage empty cartridges (water/MeOH, 100:0 to 0:100) to afford nine fractions (F01−F09) using a Selekt flash chromatography system (Biotage, Uppsala, Sweden). Among the fractions obtained, F05 (1.55 g) was further fractionated employing a flash chromatography system using a 400 g Biotage Sfär C_18_ D cartridge (water/MeOH, 60:40 to 0:100) to obtain compound 5 (122.0 mg) along with 14 subfractions (F0501−F0514). F0506 (56.1 mg) and F0512 (23.3 mg) were purified using a Waters preparative high-performance liquid chromatography (HPLC) system consisting of a 2545Q gradient module and a 2998 Photodiode array (PDA) detector (Waters, Milford, MA, USA) with a YMC-Pack ODS-A semi-preparative column (21.2 × 250 mm, 5 μm, 120 Å, water/CH_3_CN, 98:2 to 90:10, 6 mL/min, YMC, Kyoto, Japan) to yield compounds 2 (3.4 mg) and 3 (2.9 mg) from F0506 and compounds 1 (2.2 mg) and 4 (3.1 mg) from F0512. Compound 6 (7.4 mg) was also obtained after preparative HPLC using a YMC-Pack ODS-A semi-preparative column (water/CH_3_CN, 75:25 to 60:40, 6 mL/min) from F08 (40.1 mg). NMR spectra of isolated compounds were acquired using the AVANCE III 600 MHz Cryo-NMR equipment (Bruker, Rheinstetten, Germany).

### 4.11. UPLC Analysis

The PBWE (11.0 mg) was dissolved in 0.2 mL of distilled water, loaded onto a Diaion HP-20 column (10 × 35 mm), and washed out with 5 mL of water to remove non-ultraviolet-absorbing components. Thereafter, the remaining residue was washed with 5 mL of MeOH, and the extract was concentrated to finally obtain an analytical sample (1.11 mg). The sample was redissolved in water at a final concentration of 2 mg/mL and filtered through a 0.2 μm membrane filter (Pall Corporation, Port Washington, NY, USA) to obtain a sample solution for UPLC analysis. Analytical UPLC was carried out on Waters Acquity H-class plus system comprising PDA eλ detector, FTN-H sample manager, and a quaternary solvent manager with a Waters CSH^TM^ C_18_ analytical column (2.1 × 100 mm, 1.7 μm, 100 Å) at 35 °C. The mobile phase was 0.05% formic acid in distilled water (A) and liquid chromatography-grade acetonitrile (B) (JT Baker, Phillipsburg, NJ, USA) with the following elution gradient: 2% B (0–3 min), 2–20% B (3–5 min), 20–50% B (5–15 min), 50–100% B (15–15.5), and 100% B (15.5–18 min). The flow rate of the mobile phase was set at 0.3 mL/min, and the sample injection volume was set at 2 μL. The ultra-violet wavelength was monitored from 200 to 400 nm, and the chromatogram for chemical profile was detected at 254 nm.

### 4.12. Statistical Analysis

The parameters for the assays except the seizure scoring were expressed as mean ± standard error of the mean (SE) values. The data of seizure scoring were reported as median of score + interquartile. Significant difference tests were performed and comparative analytical graphs were obtained using GraphPad Prism 5 (GraphPad Software, San Diego, CA, USA). The data except the seizure scoring were analyzed by one-way ANOVA followed by Tukey’s test for post-hoc analysis. The values of seizure scoring were analyzed by non-parametric test followed by a post hoc Mann-Whitney test. *p* < 0.05 was considered statistically significant.

## 5. Conclusions

In the present study, we demonstrated that pretreatment with the PBWE alleviated TMT-induced seizures and neurodegeneration in vitro and in vivo models, potentially through the antioxidative effect of the PBWE. In addition, we isolated and identified six major compounds from the PBWE for the first time and showed the possible involvement of them in the antioxidative activity. Our findings suggest the potential of the PBWE as a preventive and therapeutic agent in neurodegenerative diseases such as seizures or epilepsy.

## Data Availability

Not applicable.

## Abbreviations

TMT	Trimethyltin
ROS	Reactive oxygen species
PB	*Protaetia brevitarsis seulensis*
PBWE	PB larvae water extract
COI	Cytochrome oxidase subunit 1
DIV	Days in vitro
LDH	Lactate dehydrogenase
FJC	Fluoro-Jade C
DPPH	2,2-diphenyl-1-picryl-hydrazyl-hydrate
SDS	Sodium dodecyl sulfate
Nrf2	Nuclear factor erythroid 2-related factor 2
HPLC	High-performance liquid chromatography
PDA	Photodiode array
NMR	Nuclear magnetic resonance
UPLC	Ultra-performance liquid chromatography
SE	Standard error of mean
DG	Dentate gyrus
NAC	N-acetyl cysteine
HO-1	Heme oxygenase-1
